# Comparison between the Behavior of Low-Yield Holstein-Friesian and Brown Swiss Cows under Barn and Pasture Feeding Conditions

**DOI:** 10.3390/ani13101697

**Published:** 2023-05-20

**Authors:** Grzegorz Grodkowski, Marcin Gołębiewski, Jan Slósarz, Tomasz Sakowski, Kamila Puppel

**Affiliations:** 1 Institute of Animal Science, Warsaw University of Life Sciences, Ciszewskiego 8, 02-786 Warsaw, Poland; grzegorz_grodkowski@sggw.edu.pl (G.G.); marcin_golebiewski@sggw.edu.pl (M.G.); jan_slosarz@sggw.edu.pl (J.S.); 2 Institute of Genetics and Animal Biotechnology, Polish Academy of Science, Jastrzębiec, Postępu 36A, 05-552 Magdalenka, Poland; t.sakowski@igbzpan.pl

**Keywords:** cows’ behavior, pasture, organic production, Holstein-Friesian, Brown Swiss

## Abstract

**Simple Summary:**

Cows are usually kept in indoor housing. They receive daily feed of similar composition at fixed times. A regular daily routine means that the cows’ behavior during the day is usually similar and does not vary from day to day. Cows grazing on pasture, however, must adapt their behavior to the changing composition of the grasses. The study was conducted on 64 Holstein-Friesian (HF) cows and 54 Brown Swiss (BS) cows between August 2016 and October 2017. The animals were equipped with sensors measuring the time spent on feed intake, rumination, physical activity, and rest. In winter, the cows were fed mainly on hay, while in summer, they took forage from pasture or freshly cut forage distributed in the barn. The study also showed behavioral differences between the HF and BS breeds. HF cows, regardless of location or forage type, spent more time foraging and chewed less than BS cows. A similar relationship was observed across all the lactation groups being studied.

**Abstract:**

Cow pasturing poses many logistical and nutritional problems. Animals have more difficulty accessing pasture feed and require more time to consume the equivalent amount of dry matter compared to total mixed ration (TMR) feed from a feed table. The study was conducted during August 2016–October 2017 on 64 Holstein-Friesian (HF) cows and 54 Brown Swiss (BS) cows. All animals were equipped with CowManager sensor devices, and the cows’ behaviors were recorded: time spent on feed intake, rumination, physical activity, and rest. In winter, cows were mainly fed hay, while in summer, they took forage from the pasture or freshly cut forage provided in the barn. The study showed that the time of day had a significant (*p* < 0.001) effect on the cows’ feeding behaviors. The study also showed behavioral differences between HF and BS breeds. HF cows, regardless of the location and type of feed provided, spent more time on feed intake and chewed less compared to the BS breed. These differences were observable in all studied lactation groups. Animals were most willing to take forage two hours before sunrise and two hours before sunset and showed an increased willingness to take feed immediately after leaving the milking parlor.

## 1. Introduction

Pasture is the natural environment for dairy cattle. It is an environment where animals have the opportunity to express natural behavior, interact with other individuals, lie down in different body positions, and selectively take up pasture forage. Access to pasture also has a positive impact on animal health and increases overall welfare [1]. It has been shown that cattle housed on pasture are less likely to develop mastitis [2,3] and lameness [4]. Despite the beneficial effects animals derive from pasture, the number of farms using this method is currently declining in Europe. This is related to the intensification of both animal and crop production [5]. In Europe, the Holstein breed is still the most popular breed, with many years of selection aimed at improving traits related to milk yield. Pasture feeding, despite its many advantages, is not suitable for high-yield cows due to the relatively low energy value of green fodder compared to TMR feed. Therefore, grazing is used mainly by small farms that employ pasture as a form of cheap feeding scheme during the summer season. However, there are countries in the world that base a significant part of their dairy production on pasture grazing. Two examples are New Zealand [6] and Ireland [7]. These countries have introduced parameters into their cattle selection indexes that take into account pasture use, the seasonality of calving, and longevity. Consequently, the genetic traits of Irish and New Zealand cows are adapted to a pasture-based feeding model, as opposed to the common genotype of high-yield Holstein-Friesian cows that require intensive feeding.

As the intensity of dairy production has continued to increase over recent years, consumers have started to pay increasing attention to the welfare conditions of the animals from which the products they buy are derived. For the consumer, it is important that dairy cows are provided with the highest possible welfare, including the opportunity to go out to pasture [8]. In response to this demand, various agricultural certification schemes are becoming increasingly popular. Examples include the organic farming system [9] and Bio Suisse [10]. Having a product certified under these schemes gives the customer the assurance it has been sourced from animals that have been provided with high levels of welfare and access to pasture. Therefore, it is possible that increasing numbers of large dairy cattle farms will start using pasture grazing over the next few years. However, it should be noted that the introduction of pasture feeding involves many changes, not only in the organization of the farm itself, but also in the behavior and circadian rhythm of the cows themselves.

The behavior of dairy cows housed in a free stall barn is fairly stable; the total duration of activities spent on fulfilling basic behavioral needs per day varies from 20 to 21.5 h in a 24 h period, of which 5–5.5 h is for eating, 12–14 h is for resting (lying down), 10 h is for ruminating, 30 min is for drinking water, and 1.5 h is time spent in passageways and on other social behaviors [11]. Conditions in the barn are usually stable. Cows receive a compositionally similar feed at the same times of day, every day. Small changes in the structure of the TMR feed can result in slight increases or decreases in feed intake times, which are generally imperceptible in the overall behavior of the cows. The situation changes when the cows go out to pasture. Pasture forage is more difficult to take up, its composition changes throughout the year, and the cows are exposed to changing weather conditions.

Despite the variable conditions, and it being more difficult to intake pasture forage, cows are happy to stay on the pasture. Many works relating to behavior and welfare have highlighted the strong motivation that cows have to stay on pasture when given the choice between pasture and barn. This is not a simple relationship, and this choice is influenced by a number of factors, including weather conditions, vegetation stage, time of day, and milk yield [12,13,14]. Animals housed on pasture change their behavior compared to cows housed in barns. This is related to greater opportunities for social interaction and the different form of forage intake. Pasture fodder is more difficult for the animals to access and, therefore, intake activities tend to take longer than for the easily accessible TMR feed [15,16]. Keeping cows on pasture for most of the day can also cause changes in their circadian cycles. Cattle are crepuscular animals, showing increased activity around sunrise and sunset [17]. On pasture, the diurnal rhythm is undisturbed, unlike the conditions in the barn, where the lights are on during the winter season several hours after dark as well as before sunrise. As a result, nighttime in the barn lasts only a few hours. In addition, the presence of attendants in the barn also affects animal behavior.

It has been shown that, as the growing season progresses and the composition of the pasture sward changes, the cows’ preferences also change. Cows are most likely to take fresh spring forage, whereas, when the quality of forage deteriorates as the season progresses, cattle prefer to take the readily available and high-energy TMR feed [13]. The motivation of cattle to take in sufficient energy is very strong; hence, cows with lower productivity are more likely to stay on pasture compared to high-yield cattle, which prefer the readily available TMR feed [12]. Atmospheric conditions, and especially the temperature humidity index (THI), can also influence the cows’ preference for housing. At high THI indices (THI >72), cattle seek shelter and prefer a shaded barn, whereas, if the temperature remains within the comfort limits, cattle prefer to stay on pasture.

Failure to provide cows with opportunities to express their natural behavior can result in reduced productivity and increased stress levels in the herd [18]. Hence, careful monitoring of the animals’ circadian behavior is essential to optimize production systems [19]. Knowledge about the activities of individual cows results in valuable information about their health status. These days, cows are equipped with many different types of sensors to measure some of their specific behaviors, such as number of steps, chewing time, or time spent on feed intake. Depending on the model, these sensors are mainly used to detect estrus and lameness. There is a growing body of work demonstrating the possibility of using cow behavior monitoring for the early detection of mastitis, metabolic diseases, or heat stress [20,21]. Most of this type of research focuses on assessing the behavior of high-yield Holstein cows kept in barns, but there is little work comparing the feeding behaviors of cows of different breeds or cows kept less intensively. Companies involved in the production of monitoring systems for herd management are also increasingly implementing additional types of functionality into their equipment, including for the detection of different types of disease and alarms warning of the possibility of heat stress. Such systems usually work well under barn conditions where the cows’ behavior is comparable from day to day and any deviation from the norm can indicate the onset of a disease entity. Under pasture conditions, cow behavior is not stable and can change depending on many factors. In order to be able to implement such solutions in grazing barns, it is first necessary to know the specific behavioral characteristics of cows of different breeds in different conditions and environments. The results of the few works that describe this issue indicate that animal behavior differs significantly depending on production potential, feeding regime, and breed. O’Connell et al. [22] compared the behavior of HF cows that had high and low production potentials. They indicated that cows with high potential had shorter grazing periods, higher feed intake rates, and spent more time ruminating compared to HF cows with lower production potential. McCarthy et al. [23] showed that cows specifically selected for pasture feeding (New Zealand Friesian) grazed longer, but had lower pasture sward grazing rates compared to HF cows. Prendiville et al. [24] showed that there were differences in ruminating time between HF and Jersey (JE) breeds, with HF cows spending more time ruminating than JE cows. Failure to account for differences in the behavior of cows of different breeds housed in different environments can result in erroneous alerts generated from automated herd monitoring systems. An example of this is the difficulty in detecting heat in cows housed on pasture and associated with greater physical activity compared to cows in barn conditions [25].

The purpose of this study was to investigate the differences in the behavior of Holstein-Friesian and Brown Swiss cows under organic production conditions. Three methods of feeding were considered: pasture-based feeding, hay-based feeding in the barn, and fresh grass provided on the feed table in the barn. Demonstrating the differences and/or similarities in the daytime behavior of cows of different breeds may help to better organize production, especially in an organic system where cows usually have large areas of pasture available. It could also be the basis for further research into new systems for detecting behavioral changes in individual animals in the herd, which would contribute to the earlier detection of disease entities and thus improve the welfare of cows.

## 2. Materials and Methods

### 2.1. Location of the Experiment

The research was conducted on a certified organic, biodynamic farm in Juchowo, located in West Pomeranian Voivodship (Poland). The farm keeps Brown Swiss (BS) (about 120 head) and Holstein-Friesian (HF) (about 250 head) dairy cattle in a free-stall box system (for lactating cows) and a free-stall deep litter system (for dry cows). The average herd performance during the study was about 6500 kg of milk per standard lactation, with an average fat content of 4.21% and protein content of 3.32%. Cows were milked twice a day on a Happel 2 × 16 herringbone-type milking parlor. The morning milking started around 5:00 a.m., and afternoon milking started at 4:00 p.m. Cows were admitted to the hall according to their associated lactation group, starting with the group that were in the first stage of lactation. The parlor was equipped with built-in ICAR-certified milk yield measuring equipment installed in the collection manifolds for each station. The fat and protein content information came from monthly tests conducted as part of the herd’s milking performance evaluation carried out by the Polish Federation of Cattle Breeders and Milk Producers.

**Figure 1:**
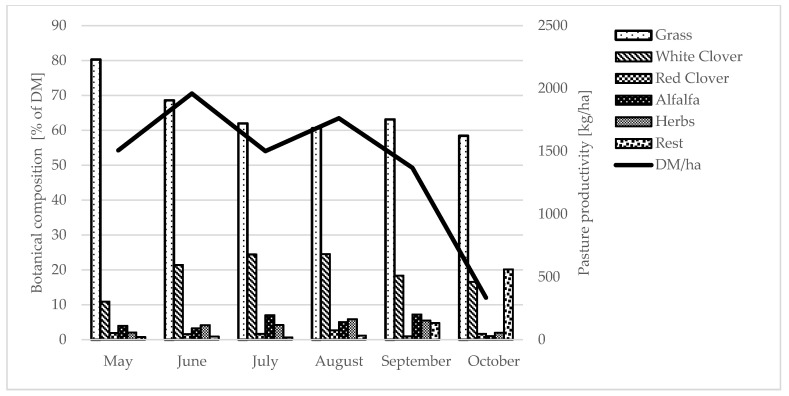
Botanical composition and productivity of pastures.

The way in which the animals were fed depended on the season. During the winter, it was based on *ad libitum* hay with the addition of concentrated feed and vitamin and mineral supplements. Hay distributed on the feed table was not crushed or mixed with other feeds. During the grazing season, whenever possible, the animals went out to pasture to take in pasture fodder. The cows were grazed on pastures in a rotational system with technological grouping, taking into account the stage of lactation. The pasture stocking rate was about 10 cows per hectare. The botanical composition of the pastures and their productivity is shown in Figure 1. The pastures were distributed on slightly undulating terrain with hills whose relative heights did not exceed 5 m. The time spent on the pasture depended on weather conditions (temperature and rainfall). When conditions were favorable, the animals spent approximately 20 h a day on pasture, going down to the barn only for milking. After milking, they received a concentrated feed supplement, the amount of which depended on the cows’ stage of lactation (from 6 to 8 kg at the beginning of lactation, 3 to 4 kg during the middle phase of lactation, and 1 kg at the end of lactation—feed concentrate per cow per day). The concentrated feed ration consisted of oats, lupine, and barley, and mineral additives in the form of sea salt, fodder chalk, and mixed vitamins and minerals. The feed concentrate was divided into two portions: one given after the evening milking and the other after the morning milking. In cases where the weather conditions were unfavorable, the animals were fed with cut, green fodder (which had not been crushed or mixed with other feed) delivered to the feed table in the barn.

### 2.2. Data Collection

The experiment began in August 2016 and lasted until October 2017. From the herd, 118 cows (64 HF breed and 54 BS breed) were randomly selected. The selected animals varied in age and stage of lactation. Group size data are presented in Table 1. The body condition score (BCS) was assessed using the BCS-5 method described by Edmonson et al. [26]; the average BCS for the cows was 3.0–3.4. During the study, the cows were under veterinary care.

The animals were fitted with CowManager sensor devices from Agis Automatisering BV, Harmelen, the Netherlands. The sensors were installed according to the manufacturer’s recommendations on the left auricle. The sensors measured cows’ activity using accelerometer-measured acceleration in three directions (3D). The data were then transmitted to a router and then further to the system provider’s servers, where an algorithm classified the data into individual activities (feed intake, rumination, rest, low physical activity, high physical activity). While the animals were in the pasture, out of range of the router, the data were stored in the device’s memory and exported to the Internet during milking in the milking parlor where the router was located.

At the start of the experiment, this was still a new system, and there were no papers available in the scientific literature dealing with the validation of this device. Therefore, before starting data collection, the system was calibrated to adjust its sensitivity to the specifics of the herd. This consisted of making visual observations of a selected cow’s behavior at one-minute intervals over a period of one hour and comparing them with data from the CowManager system. After 56 h of observations, covering different individuals, the following coefficients of determination were obtained, which allowed for the concordance between the questionnaire’s records and the system readings: feed intake R2 = 0.86, rumination R2 = 0.97, and rest R2 = 0.94 [27]. It was assumed that the system was sufficiently accurate in classifying the different types of cow behavior. Currently, other works validating this system also confirm its applicability to this type of research [28,29,30].

The height of the pasture sward during the summer season was measured once a week using a Jenquip EC10 Platemeter, with measurements being taken using the envelope method at every 10 m. A minimum of 30 measurements were taken for each pasture each time, from which the average height of the pasture sward was drawn. In order to determine the amount of dry matter in the pasture grass, a representative sample of grass was taken every two weeks from each of the pastures and meadows used in the experiment. Determination of forage dry matter content was carried out by drying it at 70 °C. The analysis of the dry matter content in the hay given to cows during the winter period was carried out in the same manner.

The results obtained from the CowManager sensors were grouped into packets, each corresponding to a particular hour. The data packets (1 packet = 1 h of measurement) contained the total number of minutes spent by the cow on a particular activity per hour. The data were additionally supplemented with, among other things, information on the various types of disease entities that were detected by the veterinarian. Information concerning lameness and the occurrence of estrus were also included in the created database. As a result of the experiment, which ran from August 2016 to October 2017, more than 960,000 data records were collected.

### 2.3. Statistical Analysis

Due to the significant behavioral changes that occur during the onset of various disease entities, estrus, and the periparturient period, such animals were not included in the statistical analysis. Data collected during the two transition periods from winter to summer feeding and the transition from pasture to winter feeding were also excluded from the analysis. During these periods, the time spent by the animals on pasture was gradually lengthened (in spring) and gradually shortened (in autumn). Any frequent changes in the feed ration during these periods could affect the behavior of the cows; hence, these data were not included in the statistical analysis. The final analysis included 181 days of winter feeding, 108 days of pasture feeding, and 64 days of grass feeding in the barn. Excluded from the analysis were 24 days of grass feeding in the barn during rainy days and 75 days of transitional feeding.

In order to analyze the overall behavior of cows in different lactation phases and feeding regimes, hourly data were summed to obtain the total amount of time spent performing a given activity by a single animal per day (min/day). Complete data were used to analyze the effect of milking time on cow behavior.

Statistical analysis was carried out using IBM SPSS 23 software. A mixed model with multiple variables was used for the calculations.

Y*_ijk_* = μ + A*_i_* + B*_j_* + C*_k_* + (A*_i_* × B*_j_*) + (A*_i_* × C*_k_*) + (B*_j_* × C*_k_*) + (A*_i_* × B*_j_*× C*_k_*) e*_ijk_*

where Y*_ijk_* is the dependent variable, A*_i_* is the breed effect (where *i* = 1 or 2, in which 1 = HF, 2 = BS), B*_j_* is the feeding effect (where *j* = 1–3, in which 1 = winter feeding, 2 = pasture season, and 3 = fresh grass in the barn), and C*_k_* is the lactation stage effect (where *k* = 1–3, in which 1 = first stage of lactation, 2 = middle stage of lactation, and 3 = last stage of lactation); (A*_i_* × B*_j_*) is the fixed interaction effect between breed and feeding; (A*_i_* × C*_k_*) is the fixed interaction effect between breed and stage of lactation; (A*_i_* × B*_j_* × C_k_) is the fixed interaction effect between breed, feeding, and stage of lactation; and e_ijkl_ is the random error.

The triple interaction was not cosidered in the results as it was not significant. The analysis of the covariance structure presented the UN structure as the covariances between the repeated measurments below and above the diagonal. Spearman’s correlation was used to evaluate the relationship between the amount of dry matter in the feed ration and the time spent on intake. For multivariable comparison, Fisher’s LSD test was applied.

## 3. Results

The analysis of the effects of breed and diet on cow behavior and milk quantity and composition showed significant differences. BS cows had higher yields compared to HF cows in terms of both milk quantity and fat and protein content (Table 1). The high standard deviation numbers are due to a failure to include the effect of the lactation phase in the analysis. This was due to insufficient data on the composition and quantity of the obtained milk.

The behavioral differences between HF and BS cows for all the analyzed feeding scenarios were also statistically significant (*p* < 0.001). Regardless of the feeding season, cows of the HF breed spent more time ruminating compared to the BS breed. In contrast, the BS breed spent more time on feed intake compared to HF cows. Activities related to physical activity and inactivity for both breeds were also statistically significant, but the differences found in chewing were not as large as those for rumination and feed intake. Nevertheless, it was shown that during pasture feeding, the HF breed cows showed more activity compared to the BS breed cows. The opposite situation was observed during winter feeding, where higher locomotor activity was observed in BS cows (Table 2).

The dry matter content of pasture green fodder and grass provided in the barn varied throughout the year from 14% to 20% and depended on the weather conditions and vegetation stage. Hay fed to cows during the winter period had between 88% and 90% dry matter. The correlation coefficient between the feed’s dry matter content and time spent by cows eating was −0.382 (*p* < 0.001).

**Figure 2:**
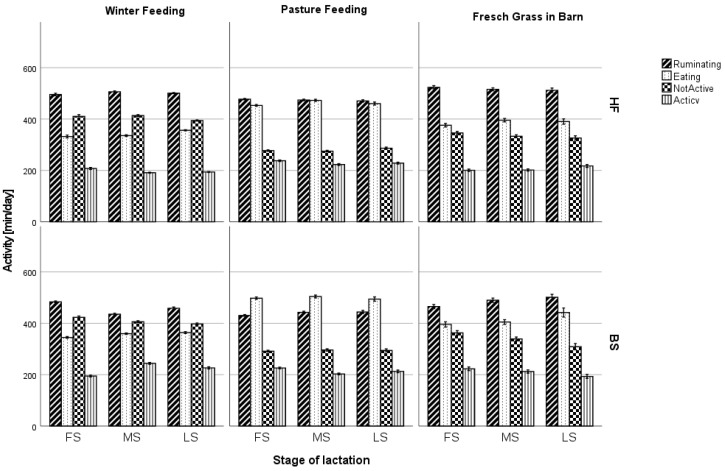
Effect of breed, feeding regime, and stage of lactation on cow behavior. FS—first stage of lactation, MS—middle stage of lactation, LS—last stage of lactation, HF—Holstein-Friesian, BS—Brown Swiss. Data are presented as least squares means with a standard error of the mean. Statistical differences between cow breed groups at *p* < 0.001, stage of lactation at *p* < 0.001, and feeding system at *p* < 0.001.

Comparing groups of cows at different stages of lactation also showed significant differences in behavior by breed and feeding season (*p* < 0.001) for all the analyzed groups. Time spent on feed intake during winter feeding increased as the lactation stage progressed. Animals in the final stage of lactation spent an average of 19 min longer at the feed table for the BS breed and 21 min for the HF breed, when compared to cows in the initial stage of lactation. A similar relationship was observed when cows were fed fresh grass in the barn. During pasture feeding, the differences between groups were insignificant (Figure 2).

**Figure 3:**
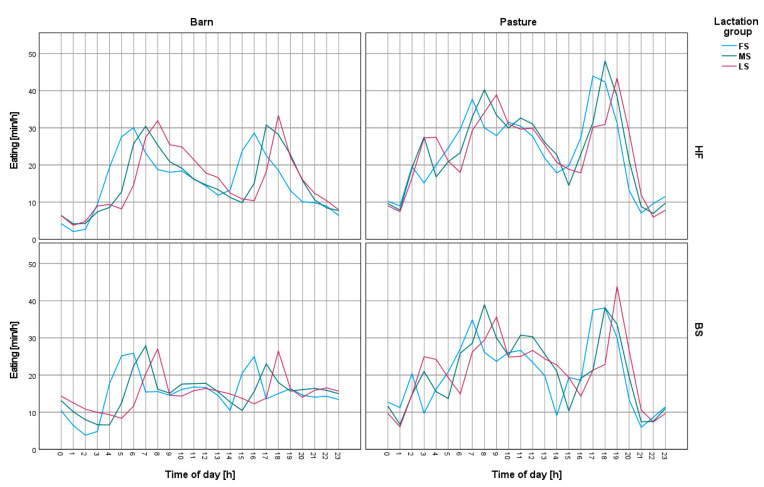
Changes in feed intake intensity on a daily basis for Holstein-Friesian and Brown Swiss cows on pasture and in the barn. FS—first stage of lactation, MS—middle stage of lactation, LS—last stage of lactation, HF—Holstein-Friesian, BS—Brown Swiss. Morning milking started around 5:00 a.m. and afternoon milking started at 4:00 p.m. Cows were admitted to the hall according to their associated lactation group, starting with the group of cows in the first stage of lactation.

When analyzing changes in the circadian cycle, the cows were divided by breed, lactation phase, and location (pasture or barn). It was found that, both for cows housed in the barn and cows grazing on pasture, the feed intake peak occurred around one hour after milking. A similar increase in feed intake was found for both the morning and evening milking. Morning milking started at around 5:00 a.m., while afternoon milking started at 4:00 p.m. Cows entered the parlor according to their lactation grouping, starting with those at the beginning of their lactation and, hence, the shift in peak feed intake in each group (Figure 3).

**Figure 4:**
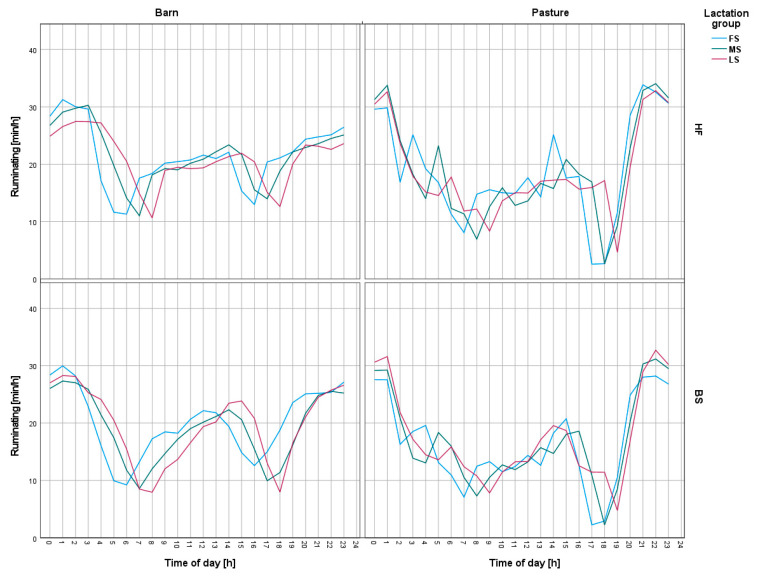
Changes in ruminating intensity on a daily basis for Holstein-Friesian and Brown Swiss cows on pasture and in the barn. FS—first stage of lactation, MS—middle stage of lactation, LS—last stage of lactation, HF—Holstein-Friesian, BS—Brown Swiss. Morning milking started around 5:00 a.m. and afternoon milking started at 4:00 p.m. Cows were admitted to the hall according to their associated lactation group, starting with the group of cows in the first stage of lactation.

A succession of increased forage intake activity can be observed around 11 a.m., after which there is a decrease in the cows’ interest in forage. Cows of both breeds, regardless of whether they were on pasture or in the barn, spent the least amount of time on feed intake in the hours before the evening milking and during the night. The influence of the night period is particularly evident in the case of cows on pasture, which between 9 p.m. and 1 a.m. hardly took up any forage at all, but, instead, spent a lot of time ruminating (Figure 4).

## 4. Discussion

The time spent by cows on individual activities can vary depending on the factors involved, for example, milking time, frequency and time of feeding [31], and the type of feed and its fineness [32,33]. In the present study, the cows’ time budgets were analyzed in relation to their diet (summer feeding—pasture, summer feeding—fresh grass in the barn, winter feeding—hay), lactation group (FS, MS, LS), and breed (HF, BS).

It is known that the time spent on pasture intake varies with the restrictions placed on the amount of time animals spend on pasture during the day. The cows’ motivation to take forage from the pasture is very strong, so reducing the time that the pasture is accessible increases the intensity of forage intake and reduces the time spent on other activities, such as resting and ruminating [34]. In this study, the focus was on analyzing natural behavior without restrictions on access to pasture. The animals studied were on pasture for approximately 20 h per day. The greatest changes in behavior were shown between cows housed on pasture with those in the barn. Pasture forage intake times averaged 460 min/day; while in contrast, the time spent by cows picking up feed from the feed table averaged 346 min/day for hay and 360 min/day for freshly cut pasture forage delivered to the feed table. This large difference between the two groups is due to the lower availability of forage on the pasture and the different physiology involved in forage intake. When cattle take forage from the pasture, they wrap their tongues around clumps of grass and tear them off. This method makes feeding increasingly difficult as the length of grass becomes shorter, resulting in cows spending more time on eating activities [35]. Fodder on the feed table can be taken in larger bites, which significantly reduces the time needed to satisfy hunger.

The results obtained in this experiment concerning the time taken by cows to take up forage correspond to some of the results obtained by other authors. Perez [35], in his work, recorded 430 min of grazing per day when access to pasture was limited to 9 h per day. Soca et al. [34] observed 486 min/day with unrestricted grazing. Other authors observed much higher values for forage intake time. RG Pulido and JD Leaver [36] obtained a figure of 541 min/day, but this experiment was conducted on pasture with a sward height of up to 9 cm. In a study by Rossi et al. [37], cows were grazed on pastures that had different yields: 2100 and 2800 kg d.m./ha. The animals spent 552 and 462 min/day on forage intake, respectively. In contrast, R. Prendiville [24], during a study on the behavior of HF and JE cows, obtained average pasture forage intake times of 648 min/day. This value is significantly higher than the study in question, especially considering the similar average productivity of the cows (about 16.9 kg/day). The authors pointed out that the increased intake time was probably influenced by the height of the pasture sward. This ranged from 10.9 to 11.4 cm before grazing and 5.3 to 4.9 cm after grazing. In the present study, the grass height oscillated around 12 cm during the summer and 7 cm at the beginning and end of the grazing season. The minimum recorded value after grazing was 5 cm, at the beginning of May and in October. These measured sward heights are similar to measurements made by McCarthy et al. [23], who reported an average pasture forage uptake time of 504 min/day, with average sward surface heights before and after grazing of 22.8 and 7.9 cm, respectively. Therefore, when comparing pasture forage intake times and pasture sward height, the results are consistent with other authors, although this parameter is highly variable and dependent on sward height and animal performance.

Cows housed in barns are usually fed with TMR feed. Many authors have carried out studies on TMR feed intake times, with very divergent results: from 168 min per day [38] to 210–300 min per day [15,39]. Nevertheless, compared to pasture-grazing cattle, cows on TMR feed spend significantly less time on feed intake. In the current study, no TMR feed was given to any of the groups under study. Cows in the winter period received a feed consisting of hay fed *ad libitum*. For this period, the average feed intake time was 346 min/day. This is longer than in the studies cited above and is probably due to the fact that feed intake time was also influenced by feed structure and dry matter content. Forage with a high content of structural fiber (NDF) is taken up more slowly than forage that is more finely ground [40]. In addition, hay tends to have a significantly higher dry matter content compared to TMR feed.

Analyzing the behavior of cows with respect to their stage of lactation showed slight increases in feed intake time as lactation progressed. This result is in contrast to that obtained by Løvendahl and Munksgaard [41], who recorded the highest feed intake times for cows in the early stage of lactation. However, it should be emphasized that the cows in the cited study were high-producing animals, which produced more than 30 kg of milk per day. In the experiment presented here, the cows produced an average of only 16 kg of milk per day, making their energy requirements, even in the early stage of lactation, much lower than high-yielding cows. It appears that high milk yields can also affect other types of cow behavior that are not related to feed intake, such as lying time and movement within the barn [42]. In contrast, a study by Munksgaard et al. [43] found no differences in feed intake time for cows at different stages of lactation with an average yield of about 26 kg milk/day.

In the present study, the average rumination time for cows on pasture was 480 min/day, which was similar to results obtained by Watt et al. [44], who, in their study, showed that cows with permanent access to pasture usually ruminated for 487 min/day. Similar results were also found in a meta-analysis by Perez-Prieto and Delagarde [16], who showed that cows grazing on pasture ruminated between 380 and 477 min/day, with a milk production above 30 kg/day. Polock et al. [45], on the other hand, in a study conducted on high-yielding HF cows, showed that the average rumination time was 330 min/day.

It has been shown that the behavior of cows is dependent on where they are housed. The behavior of the animals housed in barns can be influenced by the workers carrying out their daily duties and the associated disruption due to the light cycle. When the lighting is on, it can stimulate cows to be more active in the feed table area. In the barn where the study was conducted, daily animal handling activities typically lasted from 5 a.m. to 9 p.m., which could significantly affect natural circadian behavior. In the case of the animals that spent most of their time on pasture, the rhythm of the solar day was not disturbed by artificial lighting because, during the summer, the animals participating in the experiment went out to the pasture before sunset and returned for morning milking after sunrise. The behavior of animals in barns is also influenced by the cyclical distribution of feed. It has been proven that the moment when the feed is given is a strong stimulus for the animals to take up the feed [46].

The time of day was also significant in relation to the time at which the cows ruminated. Similar to other works [47,48], in the present study, the majority of observed ruminating time also occurred at night. As with feed intake, the cows on pasture showed greater diurnal behavior changes than cows housed in the barn.

Both HF and BS breeds are typical dairy breeds characterized by high production. However, each of these breeds have been genetically improved in different ways. HF cows were improved mainly to produce large quantities of milk in an intensive farming environment, whereas the BS breed was improved mainly to produce high-quality milk for cheese production [49,50]. The results of selection have also led to different weather condition sensitivities. It has been demonstrated that BS cows are better adapted to high temperatures compared to the HF breed [51]. The current work did not focus on evaluating the milk yield of the HF and BS breeds; however, under extensive organic production conditions, it was shown that the BS breed produced milk with significantly higher fat and protein content compared to the HF breed.

An analysis of the behavior of the two breeds in the study indicated significant differences in their behaviors according to location and diet. There are few reports in the literature on the behavior of BS and HF breeds when they are fed extensively. Braun et al. [46] showed that BS cows fed hay *ad libitum* with an average milk production of 20–25 kg spent 445 min/day on feed intake and 388 min/day on rumination. The quoted results differ from those obtained in the current experiment, in which the times for feed intake and rumination during the winter season were 350 min/day and 459 min/day, respectively. The higher feed intake time observed by Braun et al. [46] may be due to the higher productivity of the cows—as productivity increases, the average dry matter intake increases. In another study, also conducted on BS cows fed hay with silage, it was shown that cows took up feed for an average of 316 min/day [47]. This result is more in line with the forage intake times for BS cows during winter feeding in the present study (350 min/day).

Another example of a similar study is a work by Graf et al. [48], who, for BS cows that grazed all day and night on pasture, measured average forage intake times of 532 min/day and rumination times of 344 min/day, with an average yield of 17.6 kg/milk. Again, despite the similar performance of the animals, the results differ significantly from those observed in this study for BS cows on pasture feeding (499 min/day for forage intake and 436 min/day rumination). In contrast, for HF cows fed on pasture, other authors reported forage intake times averaging 522 min/day [52], that is, 497–520 min/day depending on grass height [53]. In contrast, observed rumination times averaged 464–533 min/day [53]. The HF cows in the present study spent slightly less time grazing (461 min/day) compared to the results of the other authors, while they spent a similar amount of time ruminating (475 min/day).

By comparing the two studied breeds, it was demonstrated that BS cows spent more time on feed intake and less time on rumination compared to HF cows. Similar results were obtained by Hanada et al. [54], who also studied BS cattle. Casasús et al. [55], studying the behavior of BS and Pirenaica heifers, also showed that BS animals spent significantly more time on feed intake compared to the Pirenaica breed.

## 5. Conclusions

Cow behavior is very complex and can be influenced by factors such as the structure of the supplied feed, the way in which it is supplied, animal performance, and the feeding regime. The study showed behavioral differences between HF and BS breeds. HF cows, regardless of the location and type of feed provided, spent more time on feed intake and chewed less compared to the BS breed. These differences were visible in all studied lactation groups. The presented results testify to a need to take the natural behavior of cows into account in herd management, especially when keeping animals under pasture conditions where cyclical changes in feed rations change their daily behavior.

## Figures and Tables

**Table 1:** Group size and milk yield of cows selected at the beginning of the experiment.

		First Stage of Lactation		Medium Stage of Lactation		Last Stage of Lactation	
		HF	BS	HF	BS	HF	BS
Number of animals		23	18	23	18	22	18
Milk yeld (kg/day)	LSM	22.1	24.2	15.1	14.3	8.5	9.8
	SEM	0.72	0.81	0.75	0.78	0.51	0.55

**Table 2:** Effect of breed and feeding system on milk yield and cow behavior. HF—Holstein-Friesian, BS—Brown Swiss, LSM—least squares mean, SEM—standard error of the mean, and (A*_i_* × B*_j_*) is the fixed interaction effect between breed and feeding. Means (for cattle breed in the row) marked with the same letters differ significantly at: lowercase letters, *p* ≤ 0.05; uppercase letters, *p* ≤ 0.01.

		HF			BS			*p*-Value		
	LSM /SEM	Winter	Pasture	Fresh Grass in Barn	Winter	Pasture	Fresh Grass in Barn	Breed	Feeding	Interaction (A*_i_* × B*_j_*)
Milk (kg)	LSM	12.1 ^AB^	18.3 ^A^	17.9 ^B^	15.3 ^AB^	19.8 ^A^	18.7 ^B^	<0.001	<0.001	0.143
	SEM	0.53	0.59	0.80	0.59	0.73	0.95			
Fat (%)	LSM	4.2 ^Ab^	3.65 ^A^	3.81 ^b^	4.54 ^AB^	3.93 ^A^	3.95 ^B^	<0.001	<0.001	0.140
	SEM	0.05	0.06	0.08	0.06	0.07	0.09			
Protein (%)	LSM	3.48 ^ab^	3.1 ^a^	3.1 ^b^	3.8 ^AB^	3.34 ^Ac^	3.46 ^Bc^	<0.001	<0.001	0.337
	SEM	0.03	0.03	0.05	0.03	0.04	0.05			
Ruminating (min/day)	LSM	507 ^AB^	475 ^AC^	551 ^BC^	459 ^AB^	436 ^AC^	482 ^BC^	0.000	0.000	<0.001
	SEM	0.89	0.98	1.25	1.03	1.20	1.52			
Eating (min/day)	LSM	343 ^Ab^	461 ^AC^	350 ^bC^	350 ^AB^	499 ^AC^	372 ^BC^	<0.001	0.000	<0.001
	SEM	1.05	1.16	1.48	1.22	1.42	1.80			
Not Active (min/day)	LSM	403 ^AB^	278 ^AC^	360 ^BC^	418 ^AB^	293 ^AC^	380 ^BC^	<0.001	0.000	0.179
	SEM	0.98	1.08	1.38	1.14	1.33	1.68			
Active (min/day)	LSM	192 ^AB^	231 ^AC^	213 ^BC^	218 ^a^	216 ^b^	210 ^ab^	<0.001	0.000	<0.001
	SEM	0.77	0.85	1.09	0.89	1.04	1.31			

## Data Availability

All data generated or analyzed during the study are included in this published article. The datasets used and/or analyzed in the current study are available from the corresponding author on reasonable request.
